# Extraction of Phospholipids From Crude Rapeseed Oil by *n*‐Hexane/Alcohol System: Effects of Solvent Composition on Extraction Performance and Oil Quality

**DOI:** 10.1002/fsn3.71866

**Published:** 2026-05-04

**Authors:** Fangcheng Shao, Jun Jin, Yihang Zhang, Lulu Yang, Xiuzhu Yu, Siyu Zhang

**Affiliations:** ^1^ College of Food Science and Engineering Northwest A&F University Yangling Shaanxi People's Republic of China; ^2^ State Key Laboratory of Food Science and Resources, School of Food Science and Technology Jiangnan University Wuxi China

**Keywords:** degumming, green extraction, phospholipids, rapeseed oil, structural characterization

## Abstract

Rapeseed oil contains endogenous phospholipids (PLs) and other minor bioactive components, but conventional PL extraction methods commonly rely on chloroform‐based solvent systems that are unfavorable for green and food‐related applications. In this study, *n*‐hexane/methanol and *n*‐hexane/ethanol extraction systems were evaluated as alternatives to the conventional Folch procedure for PL extraction from crude rapeseed oil. The effects of solvent type and water content on degumming performance, PL subclass composition, oil quality, and structural characteristics of the recovered PLs were systematically investigated. Compared with the Folch procedure, the *n*‐hexane/ethanol systems showed significantly improved PL extraction efficiency while maintaining high PL purity. Among the tested conditions, *n*‐hexane/ethanol (1:1, v/v) containing 7% water (HE7) gave the best overall performance, yielding 91.18% PLs with 96.54% purity, 90.18% oil recovery, and an undetectable phosphorus content. Compositional analysis showed that the recovered PL fractions were mainly composed of phosphatidylcholine (PC) and phosphatidylethanolamine (PE), although the subclass distribution varied with the extraction system. FTIR, polarized light microscopy, and scanning electron microscopy confirmed that structurally intact PL‐rich fractions were obtained and that their microstructure depended on extraction conditions. Degumming substantially reduced acid value, but also altered oxidative stability and the retention of endogenous minor components in the oil phase. Correlation and hierarchical clustering analyses further identified HE7 as the most balanced green extraction condition. HE7 represents a promising and more sustainable alternative to the conventional Folch procedure for PL recovery from crude rapeseed oil.

## Graphical abstract

Integrated analysis of extraction performance, phospholipid composition, oil quality, and multivariate relationships in green phospholipid recovery from crude rapeseed oil.

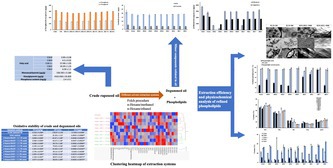

## 1 Introduction

Numerous lipid‐rich byproducts are generated during edible oil refining and are increasingly recognized as valuable resources rather than low‐value waste (Savoire et al. 2020; Zhang, Wu, et al. 2021; Smeu et al. 2022). In rapeseed oil processing, byproducts such as oil sludge and soapstock are formed during degumming and deacidification (Lu et al. 2022; Gharby 2022; Sonawane and Waghmode 2023), and these materials are notable for their relatively high phospholipid (PL) content, as well as the presence of other bioactive constituents, including phytosterols, polyphenols, and unsaturated fatty acids (Ning et al. 2023; Li and Guo 2016; Bai et al. 2022; Shen et al. 2023). Owing to this composition, rapeseed oil refining byproducts have considerable potential for high‐value utilization. However, most conventional PL extraction methods were originally developed for soybean oil, ascribed to its high PL content and wide industrial applications (Johnson and Lusas 1983).

The Folch procedure (Folch et al. 1957) and Bligh–Dyer method (Bligh and Dyer 1959) are the most widely recognized. Both methods rely on chloroform–alcohol binary systems to match the amphiphilic nature of PLs and achieve efficient extraction from complex lipid matrices. The Folch procedure uses a chloroform/methanol mixture, whereas the Bligh–Dyer method modifies the solvent composition to reduce solvent consumption and improve phase separation efficiency. These methods have provided the foundation for subsequent PL extraction protocols. Later modifications have attempted to improve operational safety and environmental compatibility through solvent substitution or the use of supercritical fluid extraction (Reverchon and De Marco 2006). Nevertheless, these developments were largely established for soybean oil or general lipid systems and were not specifically designed for the compositional characteristics of rapeseed oil refining byproducts.

This distinction is important as the rapeseed oil differs substantially from soybean oil in both PL profile and matrix composition (Robert et al. 2020; Nguyen Tuyet et al. 2014). Compared with soybean oil, rapeseed oil generally contains a higher amount of phosphatidylethanolamine (PE) and nonhydratable PLs, together with more free fatty acids (FFAs) and phenolic compounds (Lončarević et al. 2016; Wang et al. 2025). Therefore, direct application of soybean oil‐oriented extraction systems to rapeseed byproducts often leads to incomplete extraction of target PLs and reduced product purity due to co‐extraction of neutral lipids and other impurities (Carré et al. 2025; Gorka et al. 2023; Li et al. 2023). In particular, the polarity characteristics of the traditional extraction methods may not be well suited to the wider range of PL classes present in crude rapeseed oil (McClements and Gumus 2016; Drescher and van Hoogevest 2020). Moreover, the use of chloroform and methanol raises obvious concerns related to toxicity, safety, and environmental burden, which further limit the industrial applicability of these conventional methods.

Recent studies have focused on the redesign of oil extraction and refining processes using greener solvent systems that better preserve oil quality and facilitate the recovery of valuable minor constituents (Carré et al. 2025; Ribas et al. 2025). In rapeseed processing, particular attention has been paid to the fate of lipid companions and the influence of pretreatment and extraction conditions on their retention or recovery (Li et al. 2023). At the same time, broader food extraction studies have shown that green solvents, especially alcohol‐based systems, can significantly alter extraction selectivity, solvent‐solute interactions, and downstream process sustainability (Ma et al. 2025).

Among potential alternatives, *n*‐hexane/alcohol systems are of particular interest because they combine a nonpolar phase capable of dissolving the oil matrix with a more polar alcohol phase that can promote transfer of PLs and other amphiphilic components. In such systems, solvent polarity can be further regulated by adjusting the alcohol type and water content, which may substantially influence both extraction efficiency and PL selectivity. Ethanol is especially attractive from a green‐processing perspective because of its lower toxicity and greater compatibility with food‐related applications, whereas methanol can serve as a useful comparison for evaluating the role of solvent polarity in PL extraction.

Therefore, the aim of this study was to develop and evaluate green extraction systems for PL extraction from crude rapeseed oil using *n*‐hexane/methanol (HM) and *n*‐hexane/ethanol (HE) mixtures with different water contents, with the conventional Folch procedure used as a reference. The effects of solvent system on PL yield, PL purity, oil recovery, residual phosphorus, PL subclass composition, oxidative stability, minor bioactive constituents, and structural characteristics of the recovered PLs were systematically investigated. By combining direct analytical measurements with multivariate evaluation, this work was designed to identify an effective and more sustainable extraction strategy for PL recovery from crude rapeseed oil.

## 2 Materials and Methods

### 2.1 Chemicals and Reagents

Crude rapeseed oil was obtained from Qinghai Tongda Oil Processing Co. Ltd. (Xingning, China). Soybean PL mixture, β‐sitosterol, brassicasterol, and campesterol were purchased from Sigma‐Aldrich (Shanghai, China). Lutein, β‐carotene, α‐tocopherol, γ‐tocopherol, δ‐tocopherol, *n*‐octanoic acid, glyceryl monostearate, glyceryl distearate, tricaprylin, and cholesterol were acquired from Macklin (Shanghai, China). The solvents used in the present study, including *n*‐hexane, ethyl acetate, isopropyl alcohol, absolute ethanol, methanol, chloroform, and acetone, were HPLC or analytical grade and were purchased from local chemical suppliers. All chemicals were used without further treatment or purification.

### 2.2 Laboratory Degumming Procedure

A modified Folch extraction procedure (Folch et al. 1957) was used as the laboratory‐scale degumming method. Briefly, 10 g of crude rapeseed oil was mixed with chloroform/methanol/water (8:4:3, v/v/v) to a total volume of 100 mL. For comparison, two alternative extraction systems were also evaluated under the same sample loading. In these methods, 10 g of crude rapeseed oil was mixed with one of the following solvent systems, each adjusted to a total volume of 100 mL: *n*‐hexane/methanol (1:1, v/v), in which the methanol phase contained 1%–5% water, and *n*‐hexane/ethanol (1:1, v/v), in which the ethanol phase contained 5%–8% water. For clarity, the methanol‐based extraction systems are designated as HM1–HM5, corresponding to *n*‐hexane/methanol (1:1, v/v) containing 1%, 2%, 3%, 4%, and 5% water, respectively, whereas the ethanol‐based systems are designated as HE5–HE8, corresponding to *n*‐hexane/ethanol (1:1, v/v) containing 5%, 6%, 7%, and 8% water, respectively.

Degumming was carried out in a separatory funnel by repeatedly extracting the crude oil with 20 mL portions of the corresponding solvent mixture. After phase separation, the oil phase and the extracted fractions were collected separately. Solvents were then removed using a rotary evaporator (RE‐52AA, Yarong Biochemical Instrument, Shanghai, China) at 60°C, and the resulting solid residues were recovered. The *n*‐hexane and chloroform were also collected, and the degummed oil samples were collected after removal of solvent using the same rotary evaporator.

To further purify the recovered PLs, 10 mL of acetone was added to each sample, followed by centrifugation using a high‐speed refrigerated centrifuge (TGL‐16M, Hunan Xiangyi Laboratory Instrument Development Co. Ltd., Changsha, China) at 4000 rpm and −5°C for 5 min. The acetone washing step was repeated several times until the residues became white, indicating a near complete removal of residual neutral lipids and other impurities.

### 2.3 Determination of Chemical Composition of Crude Oil and PLs

#### 2.3.1 Fatty Acid Composition

Fatty acid compositions of the obtained PLs, degummed oil samples, and crude oil were determined using the AOAC Official Method 996.01 (Satchithanandam et al. 2019) on an Agilent 6890 GC system (Agilent, Santa Clara, CA, USA) equipped with an Agilent 5973 Mass Selective Detector (MSD). A VF‐17 ms column (30 m × 0.25 mm × 0.25 μm, Agilent, Santa Clara, CA, USA) was used for separating different fatty acid methyl esters, using helium as the carrier gas. Fatty acids were identified and quantified with an external 37‐FAME mixture (Supelco Inc., Bellefonte, PA, USA).

#### 2.3.2 Acylglycerols, PLs, Carotenoids, Phytosterols, and Tocopherols

A comprehensive HPLC method for the simultaneous determination of monoacylglycerols (MAGs), diacylglycerols (DAGs), PL subclass composition, tocopherols, β‐sitosterol, campesterol, lutein and β‐carotene components was developed based on previous studies (Flakelar et al. 2017; Silva et al. 2011, 2020). Analyses were performed using a Waters e2695 high‐performance liquid chromatography system (HPLC, Waters Corporation, Milford, MA, USA) equipped with a photodiode array detector (PDA) and an evaporative light‐scattering detector (ELSD, Alltech Leader Technology Co. Ltd., Chengdu, China). Separation was achieved on a Morphling WD‐SiO_2_ column (HeXi Biotechnology Co. Ltd., Manjing, China). The mobile phase consisted of three solvents: phase A, *n*‐hexane containing 2% acetic acid (v/v); phase B, ethyl acetate; and phase C, isopropanol. The gradient elution program was as follows: 0–45 min, from 100% A to 85% A/15% B; 45–65 min, linear increase to 100% B; 65–66 min, switched to 100% C; and 66–70 min, held at 100% C. The flow rate was maintained at 0.8 mL/min, the injection volume was 10 μL, and the column temperature was set at 35°C. The PDA was used to record UV spectra at the range of 190–700 nm, with 450 nm for carotenoids and 294 nm for tocopherols. Non‐UV‐absorbing compounds, including FFAs and acylglycerols, were detected by ELSD operated at 40°C with a nitrogen pressure of 3.5 bar. For sample preparation, 100 mg of sample was accurately weighed, dissolved in chloroform/methanol (1:1, v/v), and diluted to a final concentration of 10 mg/mL. The solution was centrifuged at 10,000 rpm for 5 min and filtered through a 0.22 μm hydrophobic PTFE syringe filter prior to injection. Quantification was performed using external standards, and the corresponding analytes were identified by retention time comparison with standards.

#### 2.3.3 FTIR Analysis

A Fourier transform infrared (FTIR) spectrometer of the Bruker Vertex 70 series (Bruker Optics, Rosenheim, Germany) was utilized for the identification of functional groups in this study. Approximately 0.1 g of sample was loaded directly onto a diamond attenuated total reflectance (ATR) attachment. The FTIR spectra were recorded with 32 scans, and the resolution was set to 4 cm^−1^, covering the spectral region from 4000 cm^−1^ to 400 cm^−1^.

### 2.4 Analysis of Physical Property of Crude Rapeseed Oil and PLs

#### 2.4.1 Crystalline Microstructure Analysis

The crystalline microstructure of the recovered PLs was characterized at room temperature using a polarizing light microscope (MSD‐S820, Murzider Technology Co. Ltd., Dongguan, China) and a scanning electron microscope (SEM; JSM‐6360LV, JEOL, Tokyo, Japan). For polarized light microscopy, a small amount of sample was evenly spread onto a glass slide, covered with a coverslip, and observed under polarized light at appropriate magnifications. Micrographs were recorded to evaluate crystal morphology and distribution. For SEM analysis, the samples were mounted on conductive adhesive tape, coated with platinum, and observed under 15 kV accelerating voltage with both scanning electron image (SEI) and backscattering electron image (BEI) modes to examine surface morphology and microstructural features.

#### 2.4.2 Determination of Color Parameters

Color parameters of the purified PLs and oil samples were determined using a colorimeter (3NH Technology Co. Ltd., Shenzhen, China). Before analysis, the oil samples were centrifuged at 8000 × *g* for 10 min to remove suspended impurities. The clarified samples were then transferred into 10 mm quartz cuvettes and measured under a D65 standard illuminant with a 10° observer angle. Color was recorded in the CIE Lab* color space, where L* represents lightness, a* represents the red‐green coordinate, and b* represents the yellow‐blue coordinate.

### 2.5 Evaluation of Oxidative Stability

The hydrolytic changes of the tested oils were specified by a titration method as the acid values (AV) according to the AOCS Official Method Cd 3d‐63 (American Oil Chemists' Society 2011). The primary oxidation products were determined by measuring peroxide value (PV) according to the AOAC Official Method 965.33 (American Oil Chemists' Society 2011).

Thermal oxidative stability was further assessed by differential scanning calorimetry (DSC; Setram Setline, KEP Technologies, Plan‐les‐Ouates, Switzerland). Approximately 10 mg of sample was sealed in an aluminum pan with a punctured lid, following the procedure described in a previous study (Zhang, Willett, et al. 2021) with minor modifications. Briefly, the sample was first heated from 25°C to 140°C at a rate of 30°C min^−1^ under a nitrogen flow of 50 mL min^−1^, and then held isothermally at 140°C for 6 min for stabilization. Thereafter, the purge gas was switched to oxygen at a flow rate of 50 mL min^−1^, and the sample was maintained at 140°C for 120 min. After the oxidation step, the sample was cooled from 140°C to 25°C at 50°C min^−1^ and held at 25°C for 1 min. The oxidation induction time (OIT) was determined as the onset time of the exothermic oxidation peak after switching from nitrogen to oxygen.

### 2.6 Statistical Analysis

Statistical analysis was conducted using JMP software (version 16, SAS Institute Inc., Cary, NC, USA) and SPSS Statistics (version 25, IBM Corp., Armonk, NY, USA). Data were analyzed by one‐way analysis of variance (ANOVA), and significant differences among means were determined using Tukey's honest significant difference (HSD) test at *p* < 0.05.

All experiments were conducted in triplicate, and the results were expressed as mean ± standard deviation (SD).

## 3 Results and Discussion

### 3.1 Characterization of Crude Oil

As shown in Table 1, the crude rapeseed oil used in this study exhibited a fatty acid profile typical of rapeseed oil (Chen et al. 2025), with oleic acid (C18:1) as the predominant fatty acid, followed by linoleic acid (C18:2) and linolenic acid (C18:3). Specifically, the crude oil contained 57.48% ± 1.03% C18:1, 24.39% ± 1.23% C18:2, and 6.59% ± 1.10% C18:3, whereas the contents of saturated fatty acids, including palmitic acid (C16:0) and stearic acid (C18:0), were comparatively low, at 3.99% ± 0.28% and 4.50% ± 0.32%, respectively. In addition, the crude oil contained 536.592 ± 3.184 μg/g MAGs, 4162.658 ± 35.829 μg/g DAGs, and 2.4 ± 0.1 mg/g phosphorus, indicating the presence of substantial endogenous PL‐related and partial glyceride components prior to degumming.

**TABLE 1:** Fatty acid composition, monoacylglycerol and diacylglycerol concentrations, and phosphorus content of crude rapeseed oil and degummed oils obtained using different extraction systems.

Extraction methods	Fatty acid					Monoacylglycerols (μg/g)	Diacylglycerols (μg/g)	Phosphorus content (mg/g)
	C16:0	C18:0	C18:1 c	C18:2	C18:3			
Crude rapeseed oil	3.99 ± 0.28^a^	4.5 ± 0.32^a^	57.48 ± 1.03^a^	24.39 ± 1.23^a^	6.59 ± 1.10^a^	536.592 ± 3.184^e^	4162.658 ± 35.829^e^	2.4 ± 0.1^d^
Folch procedure	3.97 ± 0.28^a^	4.57 ± 0.32^a^	57.31 ± 1.03^a^	24.32 ± 1.23^a^	6.66 ± 1.10^a^	308.367 ± 3.076^d^	3440.778 ± 184.084^b^	0.2 ± 0.0^b^
*n*‐Hexane:MeOH = 1:1 1% water	3.97 ± 0.19^a^	4.54 ± 0.30^a^	57.50 ± 1.07^a^	24.38 ± 1.14^a^	6.60 ± 1.05^a^	298.564 ± 3.945^b^	3564.223 ± 32.386^bcd^	0.3 ± 0.1^c^
*n*‐Hexane:MeOH = 1:1 2% water	4.02 ± 0.30^a^	4.53 ± 0.29^a^	57.49 ± 1.07^a^	24.36 ± 1.28^a^	6.61 ± 1.05^a^	306.311 ± 0.440^cd^	3486.424 ± 175.970^bcd^	ND
*n*‐Hexane:MeOH = 1:1 3% water	3.95 ± 0.27^a^	4.60 ± 0.34^a^	57.47 ± 0.98^a^	24.37 ± 1.18^a^	6.59 ± 1.12^a^	311.052 ± 2.198^d^	3625.609 ± 27.310^bcd^	ND
*n*‐Hexane:MeOH = 1:1 4% water	4.00 ± 0.33^a^	4.55 ± 0.31^a^	57.43 ± 1.00^a^	24.42 ± 1.26^a^	6.56 ± 1.08^a^	311.052 ± 2.198^d^	3710.934 ± 81.350^d^	ND
*n*‐Hexane:MeOH = 1:1 5% water	3.97 ± 0.25^a^	4.52 ± 0.37^a^	57.52 ± 1.11^a^	24.40 ± 1.21^a^	6.59 ± 1.14a	290.200 ± 4.218^a^	3552.153 ± 45.308^bcd^	ND
*n*‐Hexane:EtOH = 1:1 5% water	4.04 ± 0.29^a^	4.54 ± 0.28^a^	57.46 ± 1.05^a^	24.39 ± 1.30^a^	6.59 ± 1.03^a^	299.746 ± 6.273^bc^	3107.817 ± 210.382^a^	ND
*n*‐Hexane:EtOH = 1:1 6% water	3.98 ± 0.31^a^	4.57 ± 0.33^a^	57.50 ± 1.02^a^	24.38 ± 1.19^a^	6.60 ± 1.15^a^	301.020 ± 6.154^bc^	3033.431 ± 212.979^a^	ND
*n*‐Hexane:EtOH = 1:1 7% water	3.99 ± 0.26^a^	4.50 ± 0.35^a^	57.51 ± 0.99^a^	24.41 ± 1.24^a^	6.58 ± 1.06^a^	291.226 ± 4.275^a^	3683.897 ± 29.976^cd^	0.0 ± 0.0^a^
*n*‐Hexane:EtOH = 1:1 8% water	4.01 ± 0.34^a^	4.59 ± 0.30^a^	57.45 ± 1.09^a^	24.37 ± 1.22^a^	6.59 ± 1.11^a^	312.975 ± 3.554^d^	3467.263 ± 7.719^bc^	ND

*Note:* Different letters in the same column indicate values are significantly different at level 0.05 using one‐way analysis of variance (ANOVA).

Abbreviation: ND, not determined.

The initial oxidative and physicochemical properties of the crude oil also reflected its unrefined nature. The PV, AV, and OIT were 0.316 ± 0.284 mEq/kg, 0.7343 ± 0.0394 mg/g, and 178.07 ± 2.93 min, respectively (Table 2). The comparatively low PV indicates limited primary oxidation at the time of analysis, whereas the relatively high AV suggests the presence of FFAs and other hydrolytic products commonly associated with crude oil matrices. The long OIT further suggests that the native antioxidant system and original matrix composition of the crude oil contributed to relatively high oxidative stability before solvent treatment (Zhang, Willett, et al. 2021).

**TABLE 2:** Peroxide value (PV), oxidation induction time (OIT), acid value (AV), and colorimetry (L*, a*, and b*) of rapeseed oils of crude rapeseed oil and oils obtained using different extraction methods.

Extraction methods	PV (mEq/kg)	OIT (min)	AV (mg/g)	Colorimetry (Oil)		
				L*	a*	b*
Crude rapeseed Oil	0.316 ± 0.284^a^	178.07 ± 2.93^i^	0.7343 ± 0.0394^c^	26.952 ± 0.221^b^	1.257 ± 0.051^a^	2.057 ± 0.149^a^
Folch method	3.603 ± 0.095^f^	81.00 ± 1.51^g^	0.0643 ± 0.0086^ab^	24.471 ± 1.850^ab^	1.843 ± 2.154^a^	1.997 ± 3.372^a^
*n*‐Hexane:MeOH = 1:1 1% water	0.000 ± 0.000^a^	65.67 ± 1.38^ef^	0.0340 ± 0.0020^ab^	24.470 ± 2.021^ab^	1.970 ± 1.932^a^	−1.910 ± 3.248^a^
*n*‐Hexane:MeOH = 1:1 2% water	0.000 ± 0.000^a^	64.37 ± 1.30^e^	0.0347 ± 0.0023^ab^	23.690 ± 0.046^ab^	0.777 ± 0.057^a^	0.060 ± 0.121^a^
*n*‐Hexane:MeOH = 1:1 3% water	0.000 ± 0.000^a^	69.73 ± 1.86^f^	0.0493 ± 0.0090^ab^	23.657 ± 0.142^ab^	0.717 ± 0.055^a^	−0.060 ± 0.135^a^
*n*‐Hexane:MeOH = 1:1 4% water	0.105 ± 0.118^a^	79.60 ± 0.56^g^	0.0687 ± 0.0068^b^	23.617 ± 0.375^ab^	0.683 ± 0.068^a^	−0.023 ± 0.078^a^
*n*‐Hexane:MeOH = 1:1 5% water	1.341 ± 0.363^b^	91.60 ± 1.55^h^	0.0460 ± 0.0105^ab^	23.563 ± 0.374^ab^	0.757 ± 0.006^a^	−0.017 ± 0.067^a^
*n*‐Hexane:EtOH = 1:1 5% water	1.578 ± 0.079^bc^	39.13 ± 0.70^b^	0.0263 ± 0.0038^a^	23.423 ± 0.220^ab^	0.753 ± 0.086^a^	0.130 ± 0.105^a^
*n*‐Hexane:EtOH = 1:1 6% water	1.920 ± 0.166^cd^	29.30 ± 2.02^a^	0.0473 ± 0.0040^ab^	23.960 ± 0.709^ab^	0.767 ± 0.070^a^	−0.023 ± 0.023^a^
*n*‐Hexane:EtOH = 1:1 7% water	2.104 ± 0.047^d^	48.97 ± 0.15^c^	0.0557 ± 0.0071^ab^	21.400 ± 3.586a	0.930 ± 0.175^a^	0.677 ± 1.159^a^
*n*‐Hexane:EtOH = 1:1 8% water	3.077 ± 0.158^e^	58.30 ± 1.80^d^	0.0420 ± 0.0075^ab^	23.003 ± 0.458^ab^	0.713 ± 0.040^a^	0.147 ± 0.261^a^

*Note:* Different letters in the same column indicate values are significantly different at level 0.05 using one‐way analysis of variance (ANOVA).

### 3.2 Comparison of Degumming Performance Among Different Extraction Systems

**FIGURE 1:**
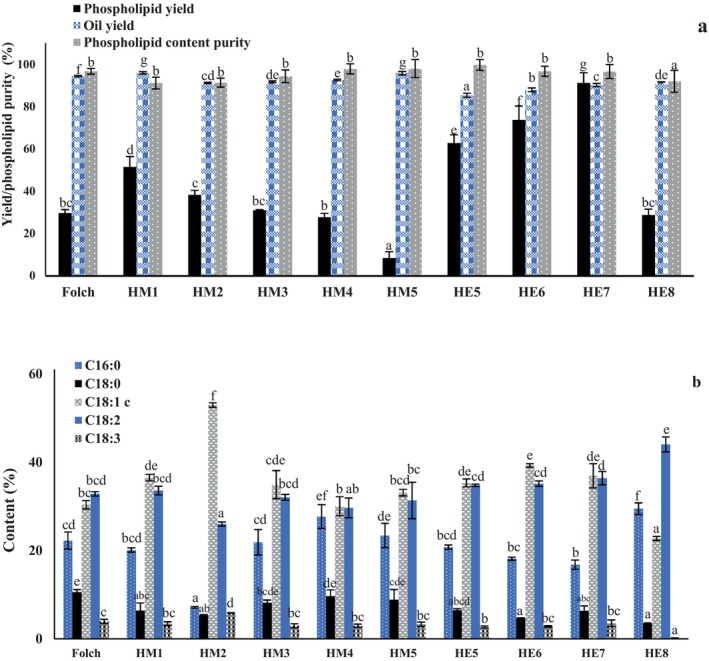
Yield and purity of the obtained phospholipids, oil recovery (a), and fatty acid composition of the obtained phospholipids (b) from Folch procedure, *n*‐hexane/MeOH (1:1, v/v) with 1% water (HM1), *n*‐hexane/MeOH (1:1, v/v) with 2% water (HM2), *n*‐hexane/MeOH (1:1, v/v) with 3% water (HM3), *n*‐hexane/MeOH (1:1, v/v) with 4% water (HM4), *n*‐hexane/MeOH (1:1, v/v) with 5% water (HM5), *n*‐hexane/EtOH (1:1, v/v) with 5% water (HE5), *n*‐hexane/EtOH (1:1, v/v) with 6% water (HE6), *n*‐hexane/EtOH (1:1, v/v) with 7% water (HE7), *n*‐hexane/EtOH (1:1, v/v) with 8% water (HE8). Different letters indicate significant statistical difference at *p*‐value < 0.05 in Tukey's honest significant difference (HSD) test.

The degumming performance of the different extraction systems was first evaluated in terms of PL yield, oil recovery, PL purity, and residual phosphorus content. Clear differences were observed among the tested solvent systems, indicating that extraction efficiency was strongly influenced by solvent composition and water content (Figure 1a). Among all treatments, HE7 gave the highest PL yield, reaching 91.18%, whereas the Folch procedure gave a substantially lower value of 29.71%. HE6 and HE5 also showed relatively high PL yields of 73.71% and 62.73%, respectively. In contrast, the HM system exhibited more variable performance, with HM1 giving 51.53%, HM2 38.25%, HM3 30.97%, HM4 27.73%, and HM5 only 8.46%. The results illustrate that the HE treatments, particularly HE7, were more effective regarding PL recovery than both the conventional Folch procedure and the HM system under the tested conditions.

Oil recovery showed a different trend from PL yield (Figure 1a). The Folch procedure and the HM system generally preserved high oil recovery, with values ranging from 91.15% to 95.90%. Specifically, HM1 and HM5 produced oil recoveries of 95.90% and 95.76%, respectively, while the Folch procedure yielded 94.35%. In contrast, the HE treatments resulted in lower oil recovery, particularly at 5% and 6% water contents, where the values decreased to 85.34% and 87.88%, respectively. HE7 showed a partial improvement to 90.18%, whereas HE8 reached 91.62%. This pattern suggests that the higher PL yield achieved by HE treatments was accompanied by greater transfer of extractable material from the oil phase, thereby reducing the mass of recovered degummed oil.

These results suggest that the extraction system must achieve a suitable polarity balance to selectively transfer PLs while minimizing excessive removal of oil‐phase components. The superior performance of the HE treatment at intermediate water content indicates that solvent composition plays a decisive role in controlling both extraction efficiency and selectivity.

The purity of the recovered PLs was high for all extraction systems, with values above 91% (Figure 1a). The highest PL purity was obtained for HE5 (99.64%), followed by HM5 (97.97%) and HM4 (97.80%). The Folch procedure also produced PLs with high purity (96.66%). HE6 and HE7 maintained similarly high purities of 96.66% and 96.54%, respectively, whereas HM1, HM2, HM3, and HE8 gave slightly lower values, although still within a comparatively narrow range of 91.10%–94.30%. The results suggest that, despite pronounced differences in extraction yield, all systems were capable of producing relatively pure PL fractions after acetone washing.

Residual phosphorus content in the degummed oils was determined for selected treatments and further reflected differences in degumming efficiency. The crude rapeseed oil contained 2.4 ± 0.1 mg/g phosphorus, whereas the Folch procedure and HM1 reduced this value to 0.2 ± 0.0 and 0.3 ± 0.1 mg/g, respectively. Among the measured treatments, HE7 showed the lowest residual phosphorus content, reaching 0 mg/g. However, for HM2–HM5, HE5, HE6, and HE8, phosphorus content was recorded as ND, indicating values were not determined rather than absent or below detection limit. Therefore, direct comparison of phosphorus removal efficiency can only be made among the crude oil, Folch, HM1, and HE7 treatments.

The fatty acid composition of the degummed oils remained largely unchanged after extraction, with C18:1 consistently remaining the dominant fatty acid, followed by C18:2 and C18:3 (Figure 1b). Across all treatments, the contents of C16:0, C18:0, C18:1, C18:2, and C18:3 showed no major deviations from those of the crude oil, suggesting that the degumming procedures selectively removed PL‐related impurities and minor polar constituents without substantially altering the fatty acid profile of the oil phase. In addition, both MAG and DAG contents were reduced after degumming relative to the crude oil, further indicating partial removal of co‐extracted polar or amphiphilic components during the extraction process.

Overall, the comparison of degumming performance showed that the HE treatments outperformed the conventional Folch procedure in PL recovery, and that water addition played a critical role in modulating extraction efficiency. Among the tested conditions, HE7 provided the best overall balance between high PL yield, high PL purity, substantial oil recovery, and nearly complete phosphorus removal, indicating that this system is a promising alternative for the green recovery of PLs from crude rapeseed oil.

### 3.3 Compositional Characterization of Recovered PLs and Degummed Oils

**FIGURE 2:**
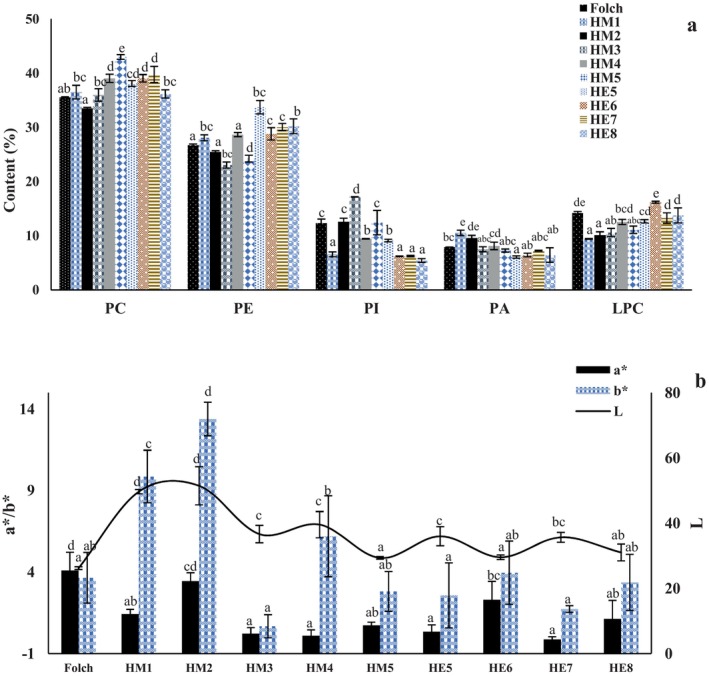
Effect of different extraction methods on the phospholipid composition (a) and color parameters (b) of rapeseed oil from Folch procedure, *n*‐hexane/MeOH (1:1, v/v) with 1% water (HM1), *n*‐hexane/MeOH (1:1, v/v) with 2% water (HM2), *n*‐hexane/MeOH (1:1, v/v) with 3% water (HM3), *n*‐hexane/MeOH (1:1, v/v) with 4% water (HM4), *n*‐hexane/MeOH (1:1, v/v) with 5% water (HM5), *n*‐hexane/EtOH (1:1, v/v) with 5% water (HE5), *n*‐hexane/EtOH (1:1, v/v) with 6% water (HE6), *n*‐hexane/EtOH (1:1, v/v) with 7% water (HE7), *n*‐hexane/EtOH (1:1, v/v) with 8% water (HE8). Different letters indicate significant statistical difference at *p*‐value < 0.05 in Tukey's honest significant difference (HSD) test.

The composition of the recovered PL fractions varied with the extraction system, indicating that solvent type and water content influenced not only PL yield, but also PL class distribution (Figure 2a). Across all treatments, phosphatidylcholine (PC) and PE were the dominant subclasses, whereas phosphatidylinositol (PI), phosphatidic acid (PA), and lysophosphatidylcholine (LPC) were present at lower proportions (Figure 2a). In the Folch‐derived PL fraction, PC, PE, PI, PA, and LPC accounted for 35.51%, 26.68%, 12.35%, 7.91%, and 14.21%, respectively, which is similar to the previous studies (Ambrosewicz‐Walacik et al. 2015; Li et al. 2023). Comparable PC‐dominant profiles were also observed for the alternative systems, although the relative proportions of individual subclasses changed noticeably among treatments. For example, HM5 yielded the highest PC proportion among the HM treatments (39.02%), whereas HE5 gave the highest PC proportion among the HE treatments (42.97%). In contrast, PE was particularly enriched in HE6–HE8, reaching 33.71%, 28.79%, and 30.03%, respectively. These results suggest the alternative solvent systems were not only effective in recovering PLs, but also selective toward different PL subclasses.

More specifically, HM treatments tended to preserve relatively balanced PC/PE ratios at lower water contents, whereas increasing water content altered the subclass distribution. HM1 and HM2 contained 36.49% and 33.49% PC, respectively, with corresponding PE contents of 28.07% and 25.48%. HM3 showed an increase in PI (17.18%), suggesting that moderate changes in solvent polarity could favor the enrichment of more polar subclasses. Within the HE treatments, HE5 was characterized by the highest PC content (42.97%), while HE7 and HE8 showed lower PC proportions but relatively stable PE contents around 30%. LPC remained within a narrower range across all treatments, although HE6 and HE8 exhibited somewhat higher LPC contents than several other treatments. Taken together, these results reveal that the solvent system can modulate PL subclass selectivity, likely through differences in polarity matching and interfacial partitioning during extraction.

The differences in PL subclass can be interpreted in terms of solvent polarity. As PL contain both polar headgroups and nonpolar fatty acyl chains, their partitioning is highly sensitive to the polarity balance between the *n*‐hexane phase and the alcohol/water phase. Adjusting the alcohol phase water content likely increased the effective polarity of the mixed solvent system and enhanced hydration of the polar headgroups, thereby affecting the selective transfer of PLs from the oil/hexane phase into the extracting alcohol phase. This may explain why moderate changes in water content altered not only total PL recovery but also the concentration of individual PL subclasses. In addition, methanol and ethanol differ in polarity and molecular association behavior, which may account for their different extraction selectivities. The HM system appeared to be more sensitive to water‐content variation, whereas the HE systems, particularly under the HE7 conditions, provided a more favorable balance between PL recovery and preservation of PC‐ and PE‐rich fractions. These results suggest that solvent‐dependent regulation of headgroup solvation and phase partitioning plays an important role in determining PL subclass composition after extraction.

**FIGURE 3:**
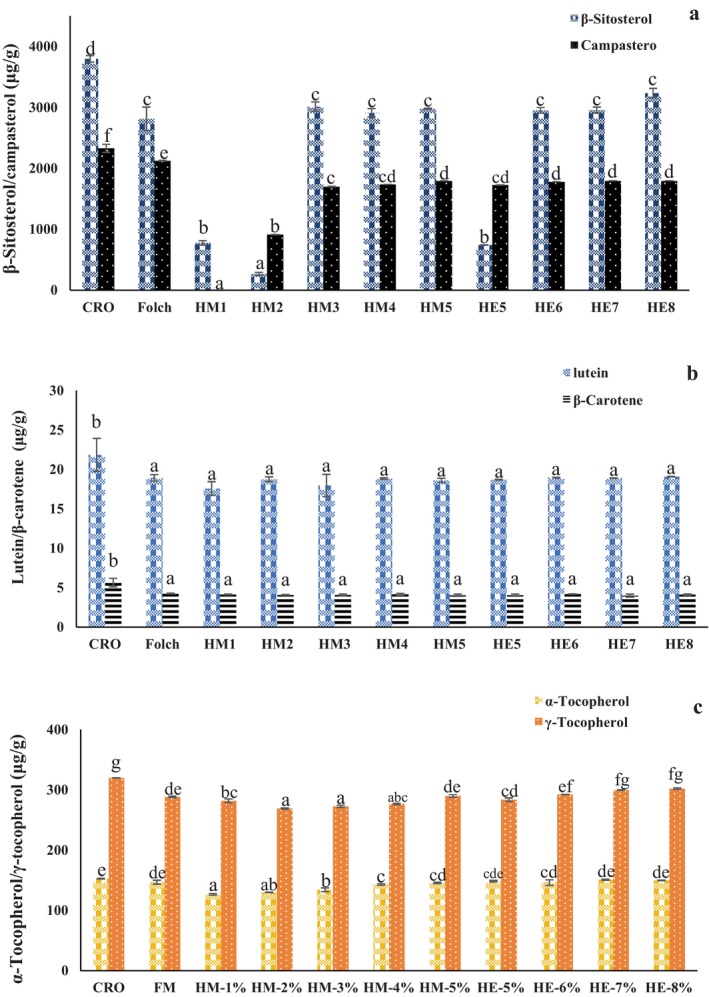
Effects of different extraction methods on β‐sitosterol and campesterol (a), lutein and β‐carotene (b), and tocopherols (c) contents of rapeseed oil from Folch procedure, *n*‐hexane/MeOH (1:1, v/v) with 1% water (HM1), *n*‐hexane/MeOH (1:1, v/v) with 2% water (HM2), *n*‐hexane/MeOH (1:1, v/v) with 3% water (HM3), *n*‐hexane/MeOH (1:1, v/v) with 4% water (HM4), *n*‐hexane/MeOH (1:1, v/v) with 5% water (HM5), *n*‐hexane/EtOH (1:1, v/v) with 5% water (HE5), *n*‐hexane/EtOH (1:1, v/v) with 6% water (HE6), *n*‐hexane/EtOH (1:1, v/v) with 7% water (HE7), *n*‐hexane/EtOH (1:1, v/v) with 8% water (HE8). Different letters indicate significant statistical difference at *p*‐value < 0.05 in Tukey's honest significant difference (HSD) test.

The compositional changes (Figure 3a) in the degummed oils were further evaluated by quantifying unsaponifiable and antioxidant‐related minor constituents, including phytosterols, carotenoids, and tocopherols. β‐Sitosterol remained the predominant phytosterol in all oil samples, but its concentration varied substantially among extraction methods. Crude rapeseed oil contained 3793.85 μg/g β‐sitosterol and 2327.03 μg/g campesterol. After extraction, β‐sitosterol decreased in most treatments, although relatively high levels were retained in HM3, HM4, HM5, HE6, HE7, and HE8, which all remained close to or above 2900 μg/g. In contrast, HM1, HM2, and especially HE5 caused much larger reductions, with β‐sitosterol contents of 776.14, 267.99, and 742.43 μg/g, respectively. Campesterol showed a similar but more variable trend, with complete loss reported for HM1 and marked reductions across most extraction conditions. These results indicate extraction solvent significantly affects sterol retention in the resulting oil phase.

Carotenoid retention also differed among treatments (Figure 3b). Crude rapeseed oil contained 21.82 μg/g lutein and 5.64 μg/g β‐carotene. After extraction, lutein decreased sharply in all degummed oils, generally to below 1.5 μg/g, indicating substantial removal or degradation during extraction and solvent handling. β‐Carotene also decreased in most treatments, although the extent of loss was smaller than that of lutein in several cases. Among the alternative systems, HE7 and HE8 showed somewhat better lutein retention than several other treatments, but the absolute concentrations remained far below that of the crude oil. These data indicate that carotenoids were more susceptible to depletion during degumming than sterols, which is consistent with their higher sensitivity to solvent environment and oxidative degradation.

Tocopherol profiles further highlighted the compositional differences among degummed oils (Figure 3c). In the crude oil, α‐tocopherol and γ‐tocopherol were present at 152.39 and 319.44 μg/g, respectively, with γ‐tocopherol being the major homolog. Following extraction, α‐tocopherol content varied widely, dropping to 126.46–150.67 μg/g in most treatments but showing a severe decrease in HM2 to 129.58 μg/g and especially HM1 to 126.46 μg/g. γ‐Tocopherol was relatively better retained than α‐tocopherol in several treatments and remained within the range of approximately 268.72–301.61 μg/g in most samples. In general, the ethanol‐based systems, particularly HE6–HE8, preserved tocopherols more effectively than the most disruptive methanol‐based treatments. This pattern suggests that although all extraction systems altered the minor‐component profile of the oil phase, ethanol‐containing systems provided a more favorable balance between PL recovery and antioxidant retention.

In summary, compositional characterization showed that the recovered PL fractions were dominated by PC and PE, but their subclass distribution was strongly affected by solvent composition and water content. At the same time, the degummed oils retained substantial amounts of sterols and tocopherols, although carotenoids were more extensively depleted. Among the tested treatments, the HE treatments, especially HE7, resulted in a favorable compositional outcome by combining high PL recovery with acceptable retention of nutritionally relevant minor constituents in the oil phase.

### 3.4 Physicochemical Properties of Degummed Oils

The physicochemical properties of the degummed oils were markedly affected by the extraction system, as reflected by changes in color (Table 2) and oxidative stability parameters. Compared with crude rapeseed oil, all degummed oils exhibited lower L* values, indicating a general decrease in lightness after solvent treatment. The crude oil showed L*, a*, and b* values of 26.950 ± 0.22, 1.257 ± 0.051, and 2.057 ± 0.150, respectively. After degumming, the L* value decreased to 24.470 ± 1.850 for the Folch procedure and further declined in several alternative systems, reaching 21.400 ± 3.590 for HE7. The a* and b* values also varied among treatments, indicating that solvent composition and water content affected not only oil lightness but also its red–green and yellow–blue tonal balance. Overall, the color changes suggest partial removal of native pigments and other minor color‐contributing components during extraction, although the extent of change depended strongly on the solvent system employed.

Among the HM treatments, the color parameters remained relatively stable across the tested water contents, with L* values ranging from 23.560 to 24.470. In contrast, the HE treatments showed greater variation, particularly HE7, where the lowest L* value was recorded. This treatment also showed a slightly elevated a* value relative to most other alternative systems. Such differences may reflect distinct extraction selectivities toward pigments, PL, and other polar minor constituents, which collectively contribute to the final visual appearance of the oil. These results imply that the solvent systems differed in their ability to remove pigments and other color‐contributing minor compounds. These color changes consequently provide indirect evidence of selective compositional redistribution during extraction.

The oxidative stability of the degummed oils was further evaluated by AV, PV, and OIT (Table 2). The crude rapeseed oil exhibited an AV of 0.7343 ± 0.0394 mg/g, a PV of 0.316 ± 0.284 mEq/kg, and an OIT of 178.07 ± 2.93 min. After degumming, the AV decreased substantially in all treatments, with values ranging from 0.0263 ± 0.0038 to 0.0687 ± 0.0068 mg/g. This pronounced reduction indicates effective removal of free fatty acids or other acid‐contributing impurities during solvent extraction. The lowest AV was observed for HE5, whereas the highest AV among the degummed oils was found for HM4. Nevertheless, all degummed samples showed significantly lower AV values than the crude oil, confirming that degumming substantially improved oil quality.

Changes in PV showed a more complex trend. The crude oil had a relatively low PV, whereas the Folch‐treated oil showed the highest value among all treatments, reaching 3.603 ± 0.095 mEq/kg. The HM system generally produced lower PVs at low water contents, with HM1, HM2, and HM3 all showing values of 0 mEq/kg. However, PV increased as water content rose further, reaching 1.341 ± 0.363 mEq/kg in HM5. The HE treatments exhibited consistently higher PVs than the low‐water methanol systems, ranging from 1.578 ± 0.079 to 3.077 ± 0.158 mEq/kg, with HE8 showing the highest PV within this group. These results illustrate that although the degumming process improved some quality attributes, certain solvent treatments may also promote the formation/retention of primary oxidation products.

OIT values also decreased substantially after degumming relative to the crude oil. The crude rapeseed oil showed the longest OIT at 178.07 ± 2.93 min, whereas all degummed oils exhibited shorter induction times, ranging from 29.30 to 91.60 min. Among the alternative systems, HM5 displayed the highest OIT, followed by the Folch procedure and HM4, whereas HE6 showed the lowest OIT. The reduced oxidation stability observed after degumming suggests that removal of PLs and endogenous minor components, including antioxidants, such as carotenoids and tocopherols, may have reduced the oxidative stability of the resulted oil, despite the concurrent decrease in AV (Rokosik et al. 2020). This interpretation is consistent with the compositional data showing partial depletion of antioxidant‐active micronutrients during extraction.

### 3.5 Structural and Microstructural Characterization of Recovered PLs

The recovered PLs were further characterized by FTIR spectroscopy to verify the preservation of their major structural features after extraction with different solvent systems. As shown in Figure S1, the spectra of PLs recovered by the Folch procedure, HM, and HE treatments exhibited broadly similar absorption profiles, indicating that the principal chemical functionalities of the PL‐rich fractions were retained irrespective of extraction route. In all samples, the bands in the 2923 and 2853 cm^−1^ regions were assigned to the asymmetric and symmetric stretching vibrations of aliphatic –CH_2_– groups from long acyl chains, whereas the strong band near 1743–1747 cm^−1^ was attributed to ester C=O stretching of esterified fatty acyl moieties. Absorptions around 1460–1465 and 1376–1378 cm^−1^ were assigned to –CH_2_– scissoring and –CH_3_ bending vibrations, respectively, further confirming the presence of lipid acyl chains in the recovered fractions.

More importantly, the PL fractions also showed characteristic absorptions in the phosphate‐related fingerprint region. Weak to moderate bands around 1236–1240 cm^−1^ can be assigned to phosphate stretching vibrations, primarily *P*=O, whereas absorptions in the 1100–1080 cm^−1^ region are consistent with P–O–C and related phosphate ester vibrations. These features, together with the persistence of strong ester and aliphatic chain bands, support the conclusion that the recovered materials were PL‐rich fractions rather than neutral‐oil residues alone. At the same time, the overall coexistence of phosphate‐region absorptions with strong hydrocarbon and ester bands is consistent with the amphiphilic molecular architecture of PLs, comprising polar phosphate‐containing headgroups and nonpolar fatty acyl chains.

Although the FTIR profiles were comparable among treatments, minor differences in relative band intensity were observed, suggesting that the extraction system influenced the detailed composition of the recovered fractions. This interpretation is consistent with the PL species determined by HPLC, which showed clear extraction‐dependent differences in the proportions of PC, PE, PI, PA, and LPC. For example, the recovered fractions remained PC‐dominant across most treatments, but PC varied from 33.49% in HM2 to 42.97% in HE5, while PE ranged from 23.03% in HM3 to 33.71% in HE5. Likewise, PI varied from 5.44% in HE8 to 17.18% in HM3, and LPC ranged from 9.42% in HM1 to 16.19% in HE6. Therefore, the subtle spectral differences among FTIR traces are reasonably attributable to compositional variation among PL subclasses and associated co‐extracted minor components, rather than to fundamental chemical degradation of the PL backbone.

For comparison, the FTIR spectrum of crude rapeseed oil was dominated by typical triacylglycerol absorptions, including weak olefinic = C–H stretching near 3006–3007 cm^−1^, strong aliphatic –CH_2_– stretching at 2923 and 2853 cm^−1^, and an intense ester carbonyl band at 1743–1747 cm^−1^ (Figure S2). By contrast, the recovered PL fractions exhibited more evident phosphate‐region features, supporting successful enrichment of polar lipids during extraction. Thus, although both crude oil and recovered PL fractions contained esterified lipid species, the FTIR data qualitatively distinguish the PL‐rich fractions from the predominantly triacylglycerol oil matrix.

**FIGURE 4:**
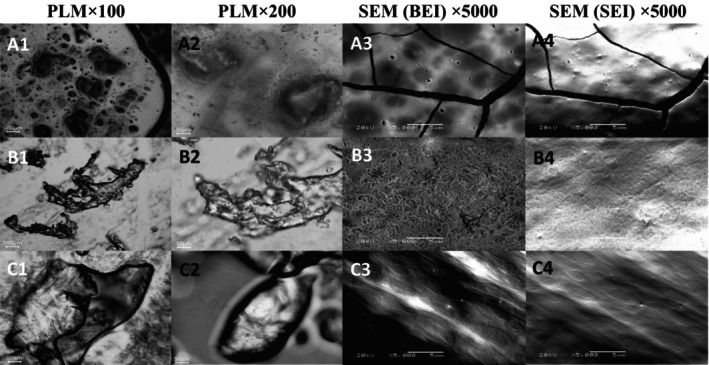
Microstructures of phospholipids recovered from crude rapeseed oil by different extraction methods, as observed by polarized light microscopy (PLM) and scanning electron microscopy (SEM, BEI and SEI modes). (A) Folch procedure; (B) *n*‐hexane/methanol (1:1, v/v) with 1% water; (C) *n*‐hexane/ethanol (1:1, v/v) with 7% water. A1–C1: PLM × 100; A2–C2: PLM × 200; A3–C3: SEM‐BEI × 5000; A4–C4: SEM‐SEI × 5000.

The microstructure of the recovered PLs was further examined by polarized light microscopy and SEM (Figure 4 and Figure S3). Representative images were provided for PLs obtained by the Folch procedure, HM1, and HE7. Under polarized light, all three samples exhibited visible ordered domains, indicating aggregation and partial structural ordering after isolation (Liu et al. 2023). However, clear differences in morphology and spatial distribution were observed among treatments. The Folch‐derived fraction showed a morphology distinct from that of HM1 and HE7, whereas the HE7 fraction, obtained under the condition giving the highest PL yield, displayed a differently organized microstructure compared with both the conventional Folch extract and the methanol‐based alternative. SEM observations further confirmed that the recovered PL fractions possessed heterogeneous aggregated structures with treatment‐dependent surface features.

The FTIR, polarized light microscopy, and SEM results demonstrate that the alternative extraction systems recovered structurally intact PL‐rich fractions while also modulating their supramolecular organization. FTIR confirmed the presence of characteristic lipid‐chain, ester, and phosphate‐related bands, verifying preservation of the essential PL framework, whereas microscopic observations revealed extraction‐dependent differences in aggregation and morphology. The results confirm the feasibility of HE‐based extraction systems, particularly HE7, as effective alternatives for PL recovery from crude rapeseed oil.

### 3.6 Overall Evaluation of the Green Extraction Strategy

**FIGURE 5:**
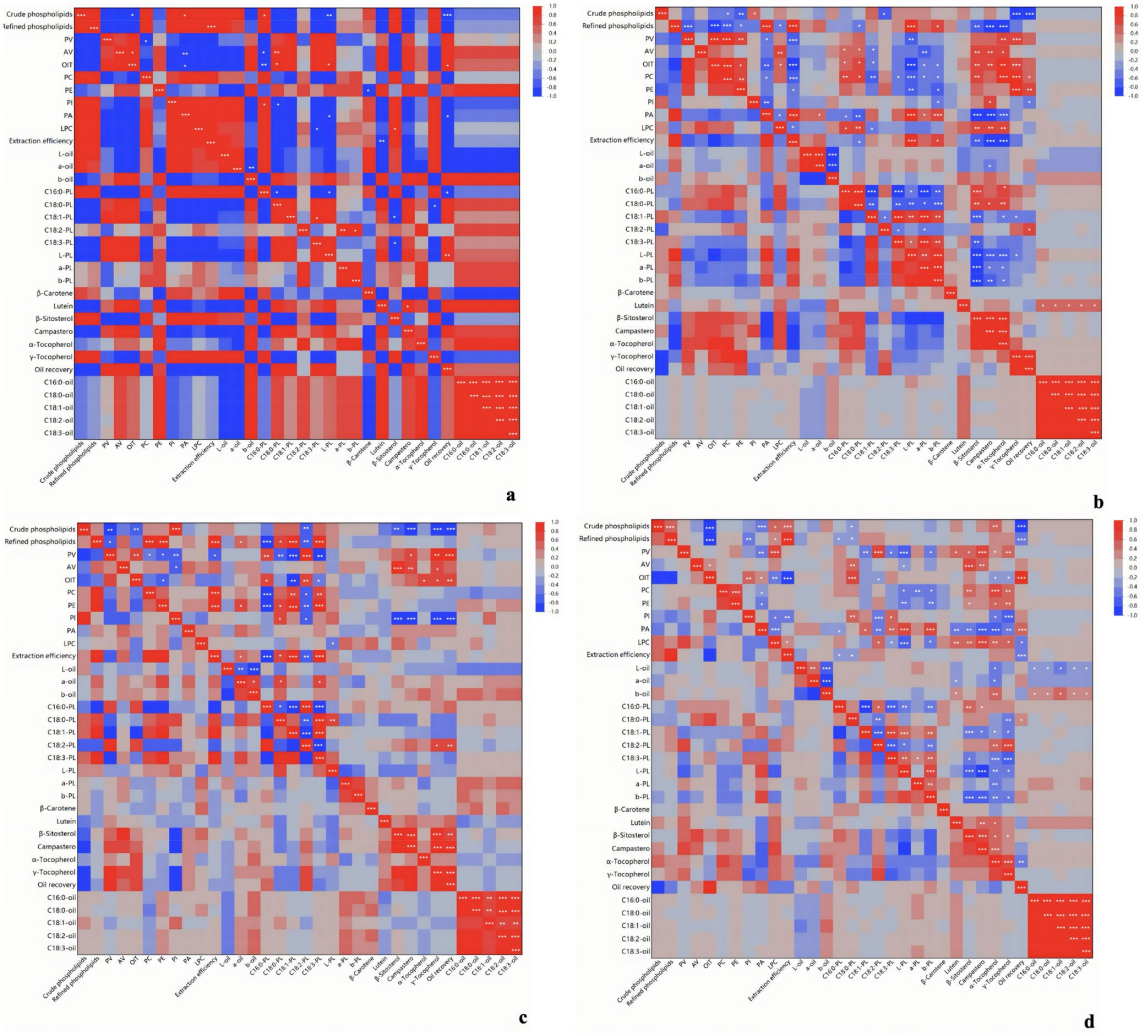
Correlation heatmap showing relationships among phospholipid yield, phospholipid composition, oxidative stability parameters, minor components, and fatty acid profiles in Folch method (a), *n*‐hexane:MeOH (b), *n*‐hexane:EtOH (c), and across all extraction treatments (d). *Indicates statistically significant at 95% confidence level; **indicates statistically significant at 99% confidence level; ***Indicates statistically significant at 99.9% confidence level.

**FIGURE 6:**
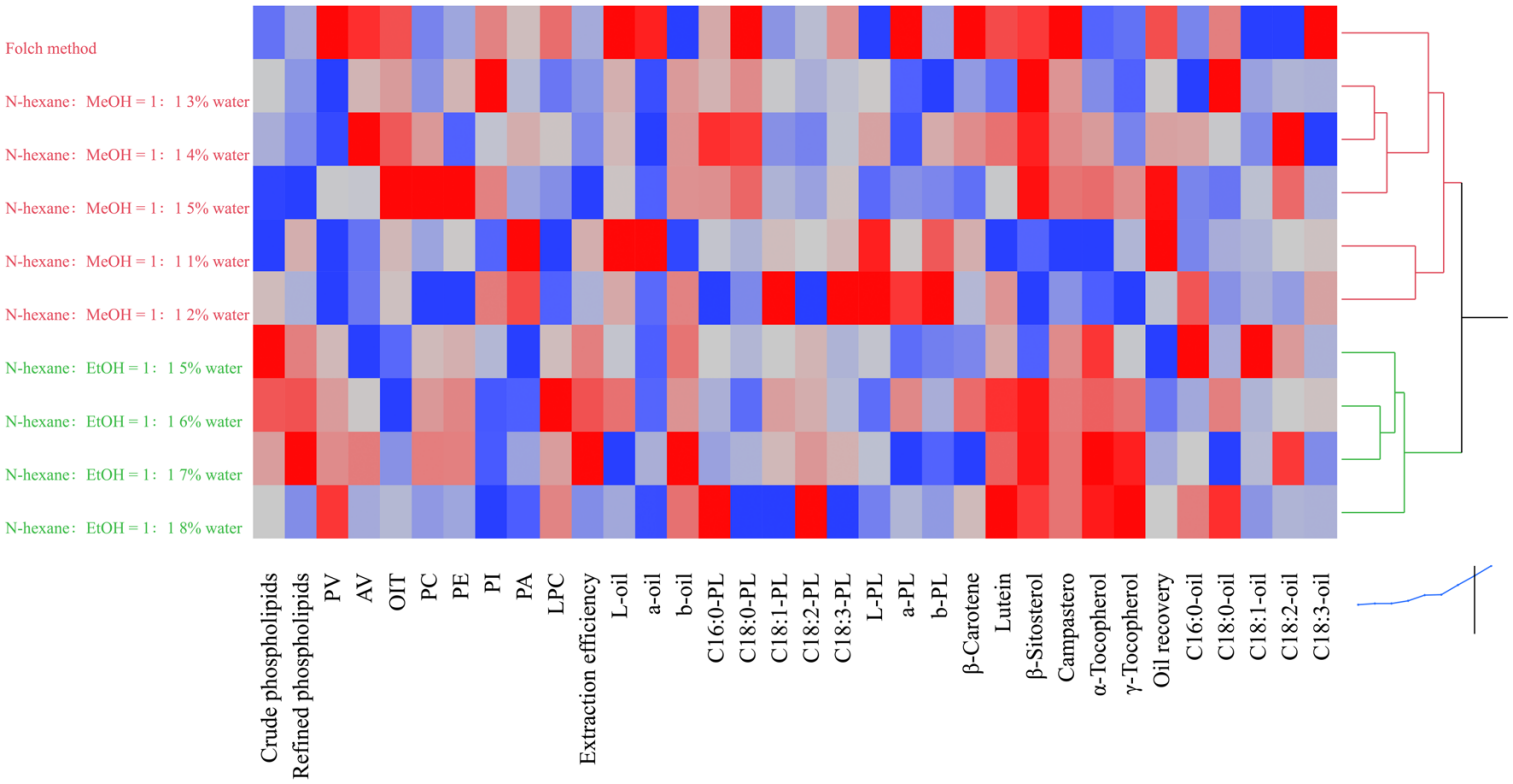
Hierarchical clustering heatmap summarizing the effects of different extraction systems on phospholipid recovery, phospholipid composition, oxidative stability, minor components, and fatty acid profiles of degummed oils and recovered phospholipids.

To further integrate the extraction results, correlation analysis and hierarchical clustering were performed using variables related to PL recovery, PL subclass composition, oxidative stability, minor bioactive constituents, and fatty acid profiles (Figures 5 and 6). In this analysis, crude PLs refer to the weight of the initially recovered PL fraction, whereas refined PLs refer to the weight of the purified PLs obtained after acetone washing. As shown in Figure 5a, the Folch procedure exhibited a relatively compact correlation structure in which crude PLs, refined PLs, and extraction efficiency were positively associated, confirming that the amount of recovered PL material was the major determinant of process performance. At the same time, these PL‐recovery variables showed differentiated relationships with oxidative stability indices and oil‐phase minor components, suggesting that PL enrichment in the Folch procedure was accompanied by compositional redistribution between the PL fraction and the degummed oil.

A different pattern was observed for the HM treatments (Figure 5b). The correlations among crude PLs, refined PLs, PL subclasses, and oil‐quality variables were more heterogeneous, indicating that changes in water content strongly affected extraction selectivity. In particular, PL‐recovery‐related variables showed pronounced associations with several PL subclasses, whereas the relationships with oil color, oxidative stability, and minor constituents were less coordinated. The results suggest that the HM treatments were sensitive to polarity adjustment, resulting in variable PL recovery behavior across the tested water levels.

By contrast, the HE treatments (Figure 5c) displayed a more coherent multivariate pattern. Crude PLs, refined PLs, and extraction efficiency were again closely associated, but the correlations with PL subclasses and several oil‐quality parameters appeared more organized than those observed in the HM treatments. This result is consistent with the direct analytical data showing that the ethanol‐based treatments, especially those containing 5%–8% water, achieved higher PL yields while maintaining high PL purity. The HE treatments therefore appeared to provide a more favorable balance between PL extraction and overall compositional control.

When all treatments were analyzed together (Figure 5d), the multivariate relationships became more clearly separated into response groups. Variables associated with PL recovery, including crude PLs, refined PLs, and extraction efficiency, formed one major block, whereas variables related to oil quality, such as PV, AV, OIT, and color parameters, occupied another region of the matrix. Minor constituents, including carotenoids, phytosterols, and tocopherols, also showed structured associations with both PL‐related and oil‐related variables. These patterns suggest that higher PL recovery was accompanied by changes in PL subclass distribution, oil physicochemical properties, and the retention of endogenous micronutrients.

The hierarchical clustering heatmap further summarized the overall similarity among extraction treatments (Figure 6). The Folch procedure and HM treatments clustered separately from the HE treatments, indicating that substitution of the HM treatments with the HE treatments significantly changed the extraction behavior. Within the ethanol‐based systems, the 5%–8% water treatments grouped closely, suggesting that ethanol‐water interactions dominated the combined responses of PL recovery, PL subclass composition, oxidative stability, minor compounds, and fatty acid distribution. By contrast, the methanol‐based treatments were more dispersed, consistent with the more heterogeneous correlation structure observed in Figure 5b.

From an industrial perspective, the HE system is more attractive than the chloroform‐based Folch procedure, as the two solvents are easier to separate and recover by conventional evaporation and condensation processes. Ethanol is also more acceptable for the food industry than methanol, ascribed to its lower toxicity. Although solvent flammability and energy needed for recovery still need to be considered in industry‐scale operation. In addition, the use of a binary HE system provides a relatively simple process structure that may be easier to integrate into existing oil refining workflows. Therefore, beyond extraction efficiency, the HE system, particularly HE7, shows potential for green and scalable PL extraction.

## 4 Conclusions

This study demonstrated that *n*‐hexane/alcohol systems can serve as effective green alternatives to the conventional Folch procedure for PL recovery from crude rapeseed oil. Extraction performance was strongly affected by solvent type and water content, affecting PL yield, subclass composition, oil recovery, and the physicochemical properties of the degummed oils. Among the tested conditions, HE7 showed the best overall performance, giving the highest PL yield, high PL purity, and satisfactory oil recovery. Although degumming substantially reduced AV, it also affected oxidative stability and the retention of endogenous minor constituents in the oil phase. Therefore, process evaluation should consider not only PL recovery, but also oil quality preservation. These results suggest that HE7 provides a promising and more sustainable strategy for PL recovery from crude rapeseed oil, with potential for the green production of PL‐rich ingredients for food and related applications. Further work is still needed to evaluate process scale‐up, solvent recovery efficiency, and optimization of the mixed‐solvent system under industrial conditions.

## Author Contributions

**Jun Jin:** writing – original draft, investigation, validation, resources, writing – review and editing. **Xiuzhu Yu:** resources, writing – review and editing. **Fangcheng Shao:** investigation, writing – original draft, formal analysis, data curation, software, methodology. **Siyu Zhang:** conceptualization, funding acquisition, writing – review and editing, project administration, supervision. **Yihang Zhang:** investigation, writing – review and editing, validation. **Lulu Yang:** investigation, writing – review and editing, visualization.

## Funding

This work was supported by the Qinghai Provincial Central Guidance Local Science and Technology Development Project (2025ZY034) and Shaanxi Provincial Department of Science and Technology (QCYRCXM‐2023‐078).

## Conflicts of Interest

The authors declare no conflicts of interest.

## Supporting information


**Figure S1:** FTIR spectra of refined phospholipids from (a) Folch procedure, (b) *n*‐hexane/MeOH (1:1, v/v) with 1% water, (c) *n*‐hexane/MeOH (1:1, v/v) with 2% water, (d) *n*‐hexane/MeOH (1:1, v/v) with 3% water, (e) *n*‐hexane/MeOH (1:1, v/v) with 4% water, (f) *n*‐hexane/MeOH (1:1, v/v) with 5% water, (g) *n*‐hexane/EtOH (1:1, v/v) with 5% water, (h) *n*‐hexane/EtOH (1:1, v/v) with 6% water, (i) *n*‐hexane/EtOH (1:1, v/v) with 7% water, (j) *n*‐hexane/EtOH (1:1, v/v) with 8% water.
**Figure S2:** FTIR spectra of (a) crude rapeseed oil and refined oil from (b) Folch procedure, (c) *n*‐hexane/MeOH (1:1, v/v) with 1% water, (d) *n*‐hexane/MeOH (1:1, v/v) with 2% water, (e) *n*‐hexane/MeOH (1:1, v/v) with 3% water, (f) *n*‐hexane/MeOH (1:1, v/v) with 4% water, (g) *n*‐hexane/MeOH (1:1, v/v) with 5% water, (h) *n*‐hexane/EtOH (1:1, v/v) with 5% water, (i) *n*‐hexane/EtOH (1:1, v/v) with 6% water, (j) *n*‐hexane/EtOH (1:1, v/v) with 7% water, (k) *n*‐hexane/EtOH (1:1, v/v) with 8% water.
**Figure S3:** PLM–PR and SEM images (BEI shadow mode) of phospholipids recovered from crude rapeseed oil by different extraction methods. (A) phospholipids recovered by the Folch method; (B) phospholipids recovered by *n*‐hexane/methanol (1:1, v/v) containing 1% water; (C) phospholipids recovered by *n*‐hexane/ethanol (1:1, v/v) containing 7% water. A1–C1: PLM‐PR × 10; A2–C2: PLM‐PR × 20; A3–C3: SEM (BEI shadow mode) × 5000.

## Data Availability

The datasets used and/or analyzed during the current study are available from the corresponding author on reasonable request.
