# Supplementary material for: Extraction of Phospholipids From Crude Rapeseed Oil by n‐Hexane/Alcohol System: Effects of Solvent Composition on Extraction Performance and Oil Quality
Source: Food Sci Nutr. 2026 May 4;14(5):e71866. doi: 10.1002/fsn3.71866 (PMC13139722; doi:10.1002/fsn3.71866)
Supplement: Supplementary file 1 — Figure S1: FTIR spectra of refined phospholipids from (a) Folch procedure, (b) n‐hexane/MeOH (1:1, v/v) with 1% water, (c) n‐hexane/MeOH (1:1, v/v) with 2% water, (d) n‐hexane/MeOH (1:1, v/v) with 3% water, (e) n‐hexane/MeOH (1:1, v/v) with 4% water, (f) n‐hexane/MeOH (1:1, v/v) with 5% water, (g) n‐hexane/EtOH (1:1, v/v) with 5% water, (h) n‐hexane/EtOH (1:1, v/v) with 6% water, (i) n‐hexane/EtOH (1:1, v/v) with 7% water, (j) n‐hexane/EtOH (1:1, v/v) with 8% water. Figure S2: FTIR spectra of (a) crude rapeseed oil and refined oil from (b) Folch procedure, (c) n‐hexane/MeOH (1:1, v/v) with 1% water, (d) n‐hexane/MeOH (1:1, v/v) with 2% water, (e) n‐hexane/MeOH (1:1, v/v) with 3% water, (f) n‐hexane/MeOH (1:1, v/v) with 4% water, (g) n‐hexane/MeOH (1:1, v/v) with 5% water, (h) n‐hexane/EtOH (1:1, v/v) with 5% water, (i) n‐hexane/EtOH (1:1, v/v) with 6% water, (j) n‐hexane/EtOH (1:1, v/v) with 7% water, (k) n‐hexane/EtOH (1:1, v/v) with 8% water. Figure S3: PLM–PR and SEM images (BEI shadow mode) of phospholipids recovered from crude rapeseed oil by different extraction methods. (A) phospholipids recovered by the Folch method; (B) phospholipids recovered by n‐hexane/methanol (1:1, v/v) containing 1% water; (C) phospholipids recovered by n‐hexane/ethanol (1:1, v/v) containing 7% water. A1–C1: PLM‐PR × 10; A2–C2: PLM‐PR × 20; A3–C3: SEM (BEI shadow mode) × 5000. [file FSN3-14-e71866-s001.docx]

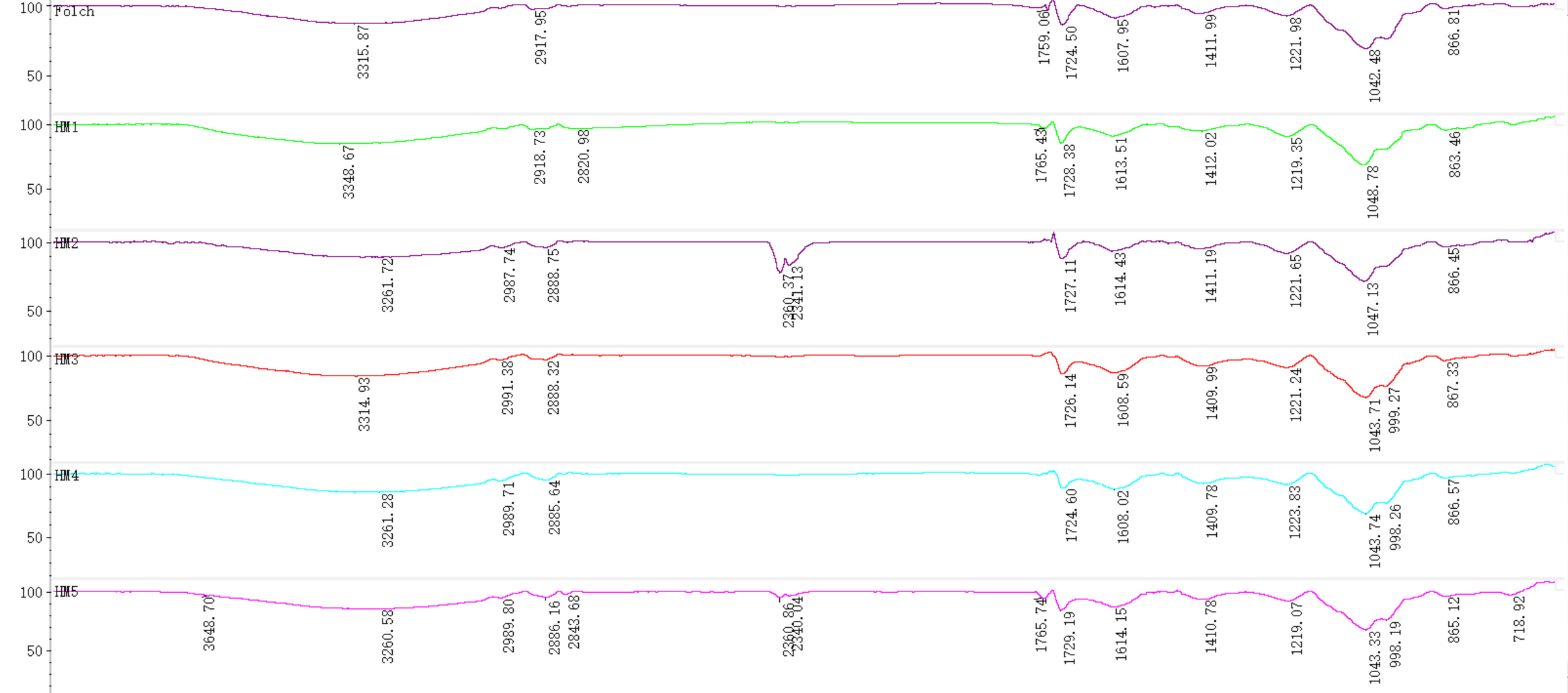

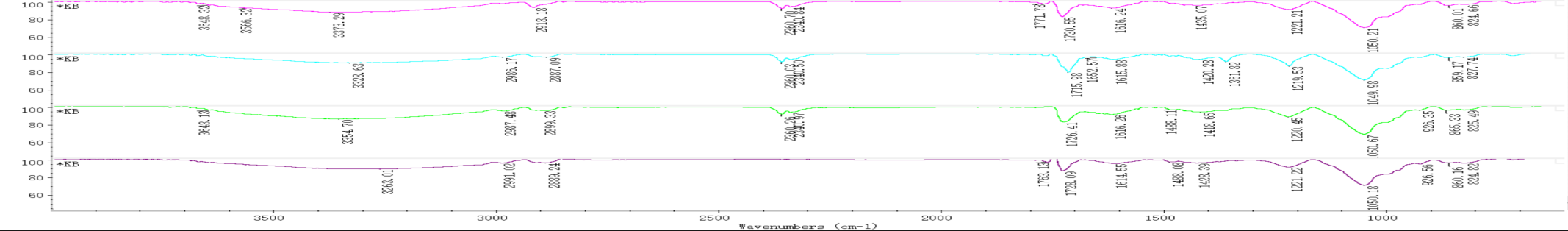


a FTIR spectra of (a) gallic acid (GA), (b) methyl gallate (MG), (c) ethyl gallate (EG), (d) propyl gallate (PG), (e) galloylglycerol (GG), (f) 1,2-dipalmitoylgalloylglycerol (DPGG), (g) 1,2-dioctanoylgalloylglycerol (DOGG), (h) coconut oil–based galloylglycerols (COGGs), and (i) coconut oil (CO).

a FTIR spectra of (a) gallic acid (GA), (b) methyl gallate (MG), (c) ethyl gallate (EG), (d) propyl gallate (PG), (e) galloylglycerol (GG), (f) 1,2-dipalmitoylgalloylglycerol (DPGG), (g) 1,2-dioctanoylgalloylglycerol (DOGG), (h) coconut oil–based galloylglycerols (COGGs), and (i) coconut oil (CO).

a

a FTIR spectra of (a) gallic acid (GA), (b) methyl gallate (MG), (c) ethyl gallate (EG), (d) propyl gallate (PG), (e) galloylglycerol (GG), (f) 1,2-dipalmitoylgalloylglycerol (DPGG), (g) 1,2-dioctanoylgalloylglycerol (DOGG), (h) coconut oil–based galloylglycerols (COGGs), and (i) coconut oil (CO).

a FTIR spectra of (a) gallic acid (GA), (b) methyl gallate (MG), (c) ethyl gallate (EG), (d) propyl gallate (PG), (e) galloylglycerol (GG), (f) 1,2-dipalmitoylgalloylglycerol (DPGG), (g) 1,2-dioctanoylgalloylglycerol (DOGG), (h) coconut oil–based galloylglycerols (COGGs), and (i) coconut oil (CO).

a FTIR spectra of (a) gallic acid (GA), (b) methyl gallate (MG), (c) ethyl gallate (EG), (d) propyl gallate (PG), (e) galloylglycerol (GG), (f) 1,2-dipalmitoylgalloylglycerol (DPGG), (g) 1,2-dioctanoylgalloylglycerol (DOGG), (h) coconut oil–based galloylglycerols (COGGs), and (i) coconut oil (CO).

b

**b** FTIR spectra of (a) gallic acid (GA), (b) methyl gallate (MG), (c) ethyl gallate (EG), (d) propyl gallate (PG), (e) galloylglycerol (GG), (f) 1,2-dipalmitoylgalloylglycerol (DPGG), (g) 1,2-dioctanoylgalloylglycerol (DOGG), (h) coconut oil–based galloylglycerols (COGGs), and (i) coconut oil (CO).

c

d

e

e FTIR spectra of (a) gallic acid (GA), (b) methyl gallate (MG), (c) ethyl gallate (EG), (d) propyl gallate (PG), (e) galloylglycerol (GG), (f) 1,2-dipalmitoylgalloylglycerol (DPGG), (g) 1,2-dioctanoylgalloylglycerol (DOGG), (h) coconut oil–based galloylglycerols (COGGs), and (i) coconut oil (CO).

f

g

h

i

j

**Fig. S1.** FTIR spectra of refined phospholipids from (a) Folch procedure, (b) n-hexane/MeOH (1:1, v/v) with 1% water, (c) n-hexane/MeOH (1:1, v/v) with 2% water, (d) n-hexane/MeOH (1:1, v/v) with 3% water, (e) n-hexane/MeOH (1:1, v/v) with 4% water, (f) n-hexane/MeOH (1:1, v/v) with 5% water, (g) n-hexane/EtOH (1:1, v/v) with 5% water, (h) n-hexane/EtOH (1:1, v/v) with 6% water, (i) n-hexane/EtOH (1:1, v/v) with 7% water, (j) n-hexane/EtOH (1:1, v/v) with 8% water.

a


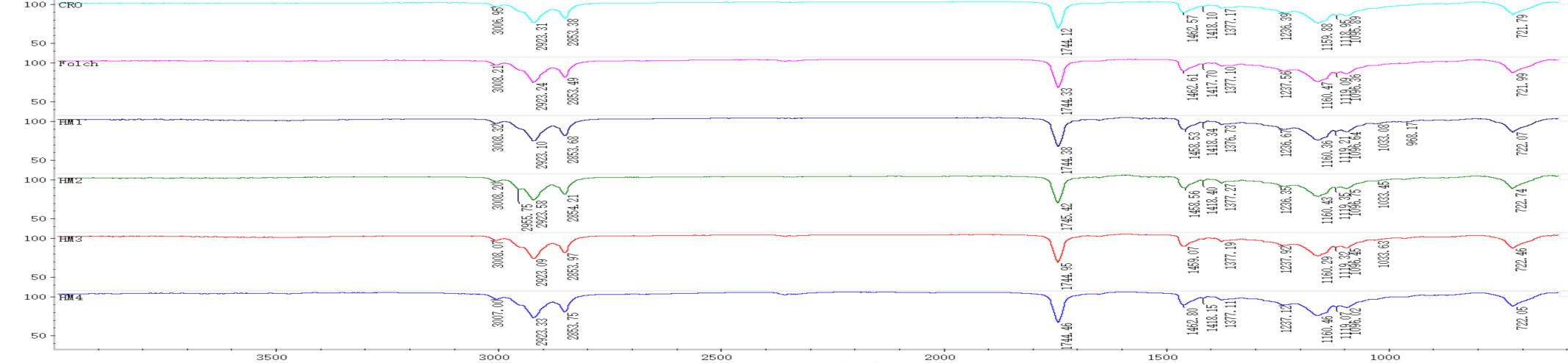

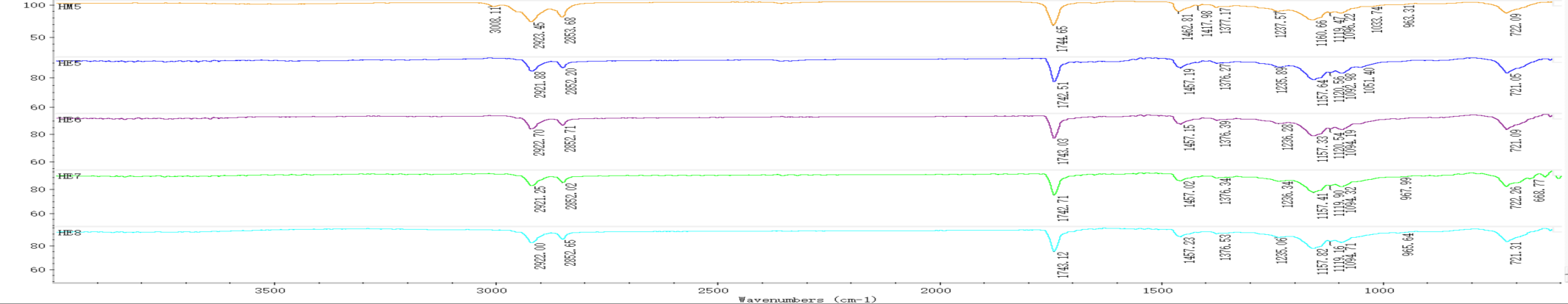


b

c

d

e

f

g

h

i

j

k

**Fig. S2.** FTIR spectra of (a) crude rapeseed oil and refined oil from (b) Folch procedure, (c) n-hexane/MeOH (1:1, v/v) with 1% water, (d) n-hexane/MeOH (1:1, v/v) with 2% water, (e) n-hexane/MeOH (1:1, v/v) with 3% water, (f) n-hexane/MeOH (1:1, v/v) with 4% water, (g) n-hexane/MeOH (1:1, v/v) with 5% water, (h) n-hexane/EtOH (1:1, v/v) with 5% water, (i) n-hexane/EtOH (1:1, v/v) with 6% water, (j) n-hexane/EtOH (1:1, v/v) with 7% water, (k) n-hexane/EtOH (1:1, v/v) with 8% water.


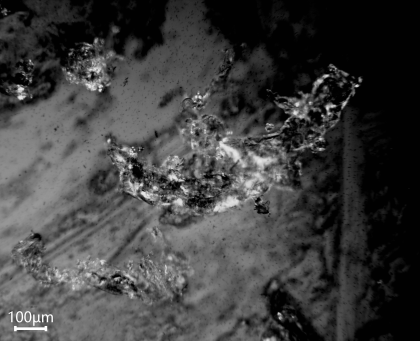

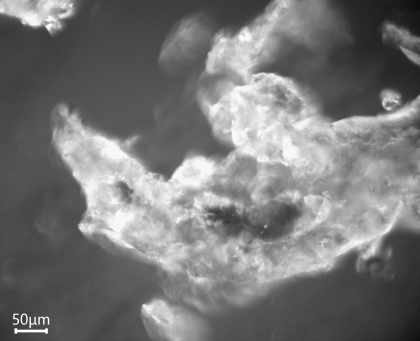

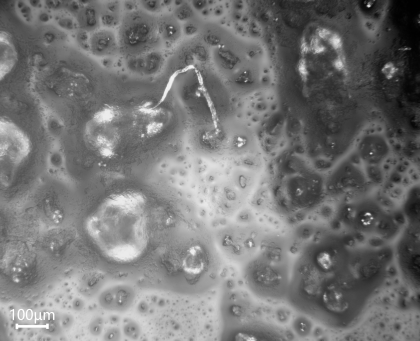

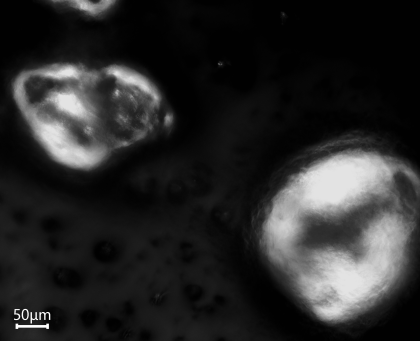

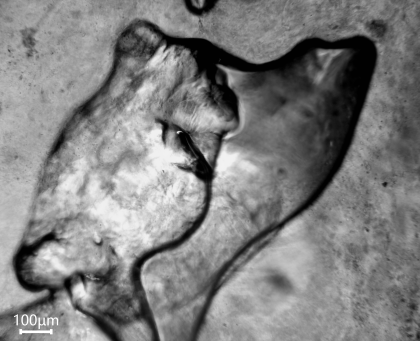

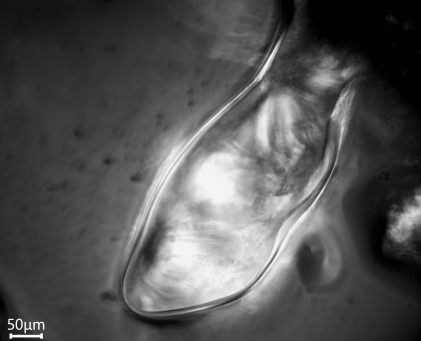

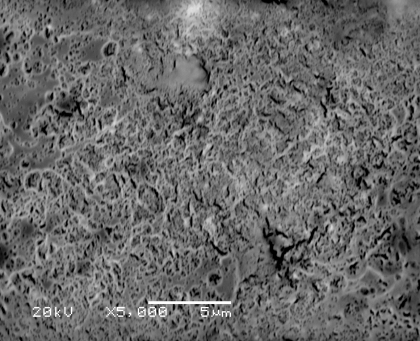

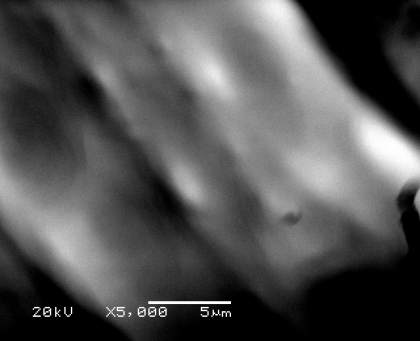

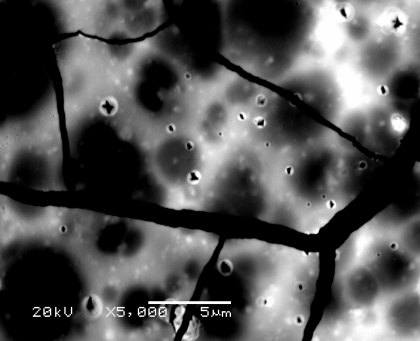


**PLM-PR×10**

**PLM-PR×20**

**SEM (shadow)×5000**

**A3**

**A1**

**A2**

**B3**

**B2**

**B1**

**C3**

**C2**

**C1**

**Fig. S3.** PLM–PR and SEM images (BEI shadow mode) of phospholipids recovered from crude rapeseed oil by different extraction methods. (A) phospholipids recovered by the Folch method; (B) phospholipids recovered by n-hexane/methanol (1:1, v/v) containing 1% water; (C) phospholipids recovered by n-hexane/ethanol (1:1, v/v) containing 7% water. A1–C1: PLM-PR ×10; A2–C2: PLM-PR ×20; A3–C3: SEM (BEI shadow mode) ×5000.
